# Machine learning algorithms for predicting PTSD: a systematic review and meta-analysis

**DOI:** 10.1186/s12911-024-02754-2

**Published:** 2025-01-21

**Authors:** Masoumeh Vali, Hossein Motahari Nezhad, Levente Kovacs, Amir H Gandomi

**Affiliations:** 1 Doctoral School of Applied Informatics and Applied Mathematics, Obuda University, Budapest, 1034 Hungary; 2 Obuda University, Budapest, Hungary; 3 Physiological Controls Research Center, University Research and Innovation Center, Obuda University, Budapest, 1034 Hungary; 4 Biomatics and Applied Artificial Intelligence Institute, John von Neumann Faculty of Informatics, Obuda University, Budapest, 1034 Hungary; 5 Faculty of Engineering and Information Technology, University of Technology Sydney, Ultimo, NSW 2007 Australia; 6 University Research and Innovation Center (EKIK), Óbuda University, Budapest, 1034 Hungary

**Keywords:** Trauma, Mental health, Model evaluation, Evidence synthesis, Deep learning, Forecasting, Artificial intelligence, Stressor

## Abstract

This study aimed to compare and evaluate the prediction accuracy and risk of bias (ROB) of post-traumatic stress disorder (PTSD) predictive models. We conducted a systematic review and random-effect meta-analysis summarizing predictive model development and validation studies using machine learning in diverse samples to predict PTSD. Model performances were pooled using the area under the curve (AUC) with a 95% confidence interval (CI). Heterogeneity in each meta-analysis was measured using I^2^. The risk of bias in each study was appraised using the PROBAST tool. 48% of the 23 included studies had a high ROB, and the remaining had unclear. Tree-based models were the primarily used algorithms and showed promising results in predicting PTSD outcomes for various groups, as indicated by their pooled AUCs: military incidents (0.745), sexual or physical trauma (0.861), natural disasters (0.771), medical trauma (0.808), firefighters (0.96), and alcohol-related stress (0.935). However, the applicability of these findings is limited due to several factors, such as significant variability among the studies, high and unclear risks of bias, and a shortage of models that maintain accuracy when tested in new settings. Researchers should follow the reporting standards for AI/ML and adhere to the PROBAST guidelines. It is also essential to conduct external validations of these models to ensure they are practical and relevant in real-world settings.

**Supplementary Information:**

The online version contains supplementary material available at 10.1186/s12911-024-02754-2.

## Introduction

A long-lasting mental disease may develop after experiencing a very stressful event such as a violent crime, a natural disaster, or severe assault and is known as post-traumatic stress disorder (PTSD) [1, 2]. PTSD is defined by symptoms that extensively persist and affect relating to others and participation in social activities. Many people with PTSD remain through unwanted, continuous memories of their traumatic event, an increased state of alertness, a tendency to avoid anything that may remind them of the trauma, and harmful patterns of thought. These symptoms of psychosis can substantially impair the quality of relationships they have in their personal lives and everyday social interactions [3]. World Health Organization (WHO) categorizes PTSD as a delayed, possibly prolonged response to a harmful, shattering incident or series of occurrences [4]. All of these will lead to significant health problems of a physical and mental nature, long-term disability, and cost to society and the individual [5]. The worldwide occurrence of PTSD in some countries has been calculated to be 3.9% [6]. In 2018, it was estimated that the overall excess economic cost of PTSD in the United States was $232.2 billion, which is equivalent to $19,630 for each person who suffers from PTSD [5]. This is of immense value, not only to the patient but to the whole healthcare system, as it allows them to start PTSD treatment on time by identifying them at an early stage. Early intervention in treating PTSD can enhance outcomes for affected individuals. Specifically, this is because treatment can commence before severe symptoms and signs appear [7]. Moreover, research indicates that treatment costs and the length of hospital stays for patients with stress disorders increase by 80%. Thus, initiating treatment early could substantially reduce the expenses related to trauma insurance [8].

With emerging artificial intelligence (AI) and machine learning (ML) technologies in recent years, an opportunity has been availed for dealing with health problems, one of which is PTSD, through improved diagnosis and prediction of diseases. However, the effectiveness of such models depends on the quality of the studies and bias management [9]. Another advantage is the integration of ML into PTSD research with a potential betterment of study characteristics for personalized medicine [10]. The studies employed ML techniques to help make sense of complex patterns in clinical and neuroimaging data and are to be applied in developing these therapeutic strategies [11]. This represents a leap to understanding and applying ML in managing PTSD [12]. However, ML promises also come with biases that might draw inappropriate conclusions and hinder its effective application in clinical practice. In addition, some sources of bias exist in data collection, algorithm design, and analysis techniques, with all their potential consequences for reducing the validity of research findings [13].

The interest in applying machine learning (ML) techniques to PTSD research is on the rise. This increasing focus shows the importance of critically examining the potential biases within these studies. Our systematic review employs the Prediction model Risk Of Bias ASsessmenT (PROBAST) tool [14], a validated instrument created to evaluate biases in research that develop predictive models. This tool plays a crucial role in ensuring the reliability and accuracy of conclusions drawn from ML studies in PTSD. This tool allows for a detailed examination of the methodological quality of ML studies and helps pinpoint areas susceptible to bias. The main goal of this review is to critically analyze the current practices in machine learning within the context of PTSD research. It evaluates the effectiveness of these studies by examining how accurately the ML models perform across different PTSD populations. Through highlighting strengths and weaknesses, this review aims to support improving predictive models, ultimately enhancing clinical practice and patient outcomes in PTSD care.

## Method

The methodology of the current systematic review and meta-analysis has been developed in accordance with the PRISMA guidelines [15].

### Eligibility criteria

The eligibility criteria for our review were articles written in English, which used machine learning techniques to predict PTSD among various populations, irrespective of gender, age, or ethnicity. We focused on peer-reviewed papers on machine learning, excluding review articles, non-English-language articles, non-peer-reviewed resources, conference papers, letters, abstracts, protocols, errata, and comments. The inclusion criteria for the predictive models under review included those describing the use of standard machine learning or deep learning techniques toward forecasting PTSD across all patient groups and demographic contexts.

### Search strategy

We searched comprehensively in three electronic databases, PubMed, Scopus, and Web of Science, for articles published in 2018 or later up to 14 December 2023. The search syntaxes for each database are detailed in S1.

### Screening and data extraction

First, studies were retrieved from each electronic database and saved in an Excel file. Duplicates were identified and removed using the DOI numbers of the articles. Titles were used instead where DOI numbers were not available. Two reviewers (M.V and H.M.N) independently screened the articles in two phases: title/abstract and full-text. Initially, the title and abstract of the studies were screened based on the eligibility criteria by both reviewers. In the next step, articles that passed the first phase underwent full-text screening, where their full texts were reviewed independently by the two reviewers. In both phases, disagreements between the two reviewers were resolved by consensus. When a disagreement arose, the two reviewers reviewed the relevant data and the inclusion/exclusion criteria to arrive at a mutually agreed decision.

The following data was extracted from the final eligible articles: the name of the first author and publication year, journal, population characteristics and their age and gender, the type of algorithm used for prediction, areas under the curve (AUCs) and their 95% confidence intervals (CIs), and other performance indicators. Subsequently, the data was analyzed and presented using descriptive statistics and cross-tabulation.

### Data analysis

We sort the reported AUCs from the studies with the following types of populations before we conduct the meta-analyses: War and military experiences, pandemic-related stress, Sexual and Aversive/Physical assaults, Medical trauma, general populations of PTSD, Commercially insured adults, Firefighters, Alcohol use and related stress. Then, within each population, we pooled AUCs from similar types of models: linear models, support vector machines (SVM), tree-based models, ensemble methods, Bayesian models, and neural networks.

To conduct a meta-analysis, at least two studies must be available to combine their AUCs. We employed qualitative evidence synthesis because a meta-analysis cannot be performed with only one AUC. AUCs were pooled separately for each population, with distinct pools for internal, external, and algorithm types using random-effect meta-analysis. AUCs were aggregated without regard to the structure of the model or its characteristics [16]. When studies did not provide the 95% CI for AUC-ROC findings, we used the following formula to calculate them [17]:

$$\:CI=AUC\:\pm\:\:{Z}_{1-\frac{\alpha\:}{2}}\:\times\:se$$

$$\:se=\:\sqrt{\frac{{q}_{1}+\left({n}_{1}-1\right){q}_{2}+\left({n}_{2}-1\right){q}_{3}}{{n}_{1}{n}_{2}}}$$

$$\:{q}_{1}=AUC\left(1-AUC\right),\:\:\:\:\:{\:\:q}_{2}=\frac{AUC}{2-AUC}-{AUC}^{2}\:,\:\:\:\:\:\:{q}_{3}=\:\frac{{2AUC}^{2}}{1+AUC}-{AUC}^{2}\:$$

Each meta-analysis employed Higgins I^2^ to assess the overall heterogeneity and variability among the AUCs. Forest plots displaying an I^2^ value greater than 50% indicate significant heterogeneity [18, 19]. Since the studies were drawn from wide-ranging populations, a random-effects meta-analysis was performed [20]. In the case of reporting the internal and the external AUCs together, the algorithm was considered an independent study [21]. The Egger test [22] was used to assess the presence of publication bias in the meta-analyses. However, according to guidelines, testing for publication bias is not recommended in meta-analyses with fewer than ten studies [23, 24]. Therefore, we did not assess publication bias in meta-analyses with fewer than ten studies. Sensitivity analyses were performed in meta-analyses, including at least three studies to evaluate the effect of any specific study on the pooled effect sizes or heterogeneity. Meta-analyses were conducted using the Medal^®^ Statistical Software, version 22.014 (MedCalc Software Ltd, Ostend, Belgium) [25]. Statistical significance was considered at a confidence level of 95%, and p-values of < 0.05 were used to denote statistical significance. Forest plots were built with the help of MedCalc software and Python.

### Risk of bias assessment

One of the tools that may be applied for conducting a critical evaluation of studies that are engaged in establishing, validating, or updating prediction models for customized predictions is the PROBAST tool [14]. A total of twenty signaling questions are included, and they are arranged into four distinct categories: participants, predictors, outcomes, and analysis. It is possible to respond to each signaling inquiry with “yes,” “probably yes,” “no,” “probably no,” or “no information.” To indicate that a domain is at high risk of bias, at least one of the signaling questions should be answered with a “no” or a “probably no.” The PROBAST checklist was used to evaluate the risk of bias and the applicability of the studies included in the analysis. Concerns about the article’s applicability and potential for bias were assessed independently by the two authors (H.M.N and M.V). A low risk of overall bias can only be evaluated once all domains have been reviewed and shown to have a low risk [26].

## Result

Three thousand forty-nine documents were retrieved from PubMed, Scopus, and Web of Science, accounting for 989, 975, and 1,085, respectively. Following deduplication, 1750 duplicates had been removed, leaving 1299 articles that underwent screening based on the established inclusion/exclusion criteria.

**Fig. 1:**
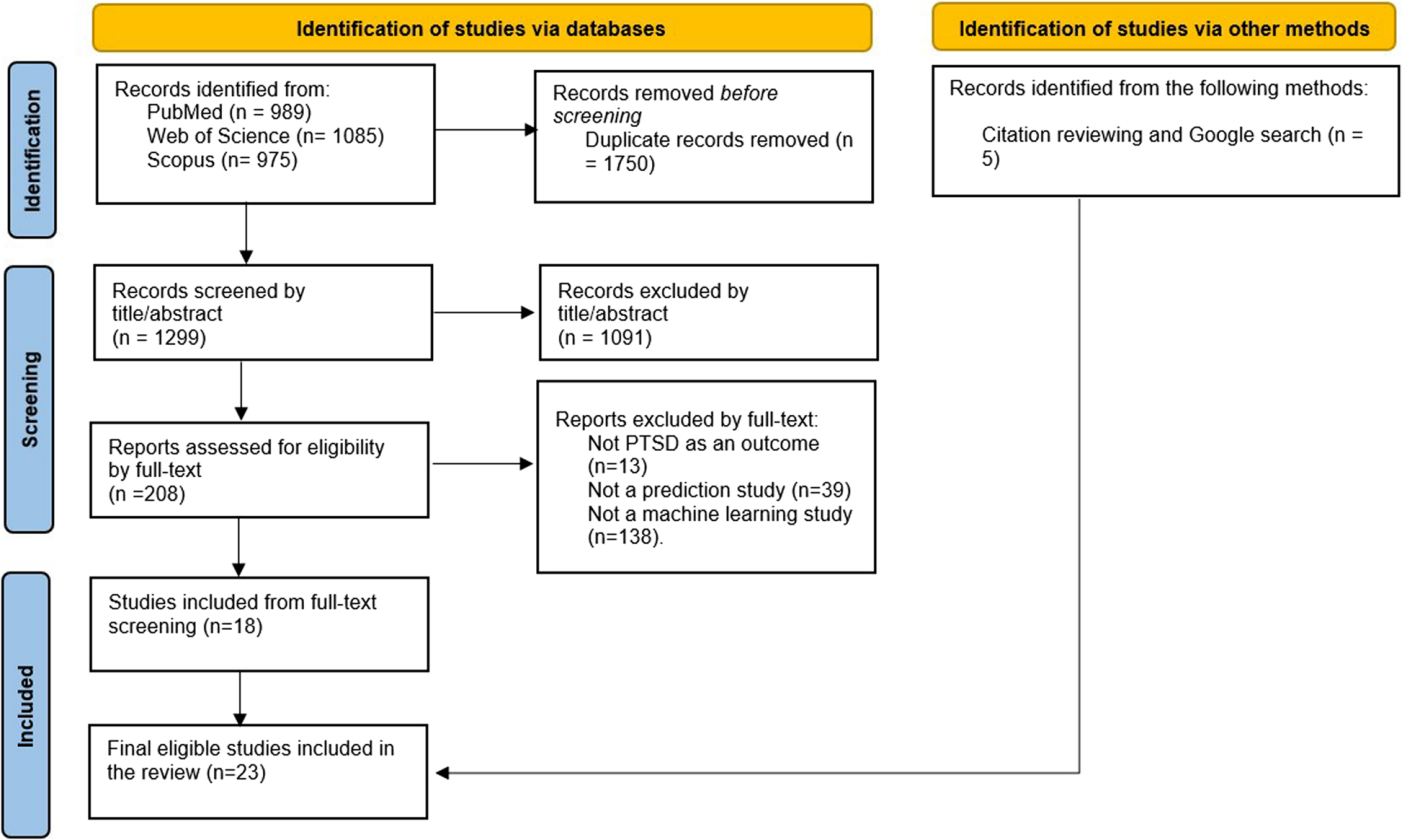
PRISMA screening

Thus, 1,091 articles were excluded after screening titles and abstracts based on a preliminary assessment that considered them irrelevant for inclusion in this review. Hence, 208 articles were considered for further evaluation through full-text screening. The screening resulted in 208 articles, which underwent full-text screening, and only 18 final studies were considered after excluding 190 ineligible for review. To improve precision and find all related studies, the reference lists of the 18 studies were also reviewed. Furthermore, a similar search using the keywords in Google resulted in five other relevant studies, totaling 23. For more details, see Fig. 1, which gives a PRISMA process of screening and selecting studies.

### Characteristics of the included studies

A significant proportion of the articles (39%, *n* = 9) were published in 2022 [11, 27–34], with 2019 [35–38] and 2021 [39–42] each contributing four articles (17%). Publications from 2018 accounted for three articles (13%) (Nicholson et al., 2018; Papini et al., 2018; Rosellini et al., 2018), while 2023 saw two publications (9%) (Dell et al., 2023; Papini et al., 2023) and one article (4%) published in 2020 [48]. Most of these articles (65%, *n* = 15) originated from researchers in the United States [11, 31–33, 35–39, 42, 44–48] and China (17%, *n* = 4) [27, 28, 30, 40]. Canadian [34, 43] and Turkish researchers [29, 41] contributed two articles each (9%). Four articles (17%) were published in the Journal of Affective Disorders [30, 35, 41, 46], while BMC Psychiatry [34, 48] and the Journal of Traumatic Stress [11, 33] each presented two articles (9% each). Additionally, two articles (9%) were published in Psychological Medicine [31, 43]. The remaining 14 articles (57%) were published in 13 journals.

The articles reviewed had various population samplings of people exposed to differing stressors to predict PTSD. Several studies focused on populations with medical trauma 26%, *n* = 6) [11, 27, 31, 39, 42, 44]. Other research works were carried out aimed at understanding the PTSD predictors in individuals exposed to sexual or physical/aversive experiences (17%, *n* = 4) [29, 32, 41, 43]. Additionally, the impact of natural disasters (13%, *n* = 3), such as earthquakes and hurricanes [40, 45, 46], was examined in specific populations. Three studies (13%) have introduced models for predicting PTSD in individuals exposed to war or military [37, 38, 47]. Three additional papers (13%) focused on the general population affected by PTSD [33, 35, 36]. The studies also included specific professional groups like pandemic-related stress (4%, *n* = 1) [28], firefighters (4%, *n* = 1) [30], alcohol use and related stress (4%, *n* = 1) [48], and insured adults (4%, *n* = 1) [34].

One study assessed the validation of a model externally that employed a gradient boosting machine method, achieving an AUC of 0.74 with 95% CIs of 0.71 to 0.77 [47]. Another article also employed both internal and external validations using extreme gradient-boosting algorithms [11]. Fourteen articles utilized tree-based models to conduct prediction studies [5, 19, 34]– [37, 21, 23, 25]– [27, 30, 31], 33]. Additionally, among the included articles, linear models were the focus of eight articles for developing prediction models for PTSD [27, 32, 33, 36, 44–46, 48]. Regarding other methodologies, SVM [35, 36, 38, 40, 45] and neural networks [28, 29, 31, 38, 40] were implemented in five articles each, ensemble methods in three [36, 39, 45], and Bayesian models [35, 43] In two studies. Table 1 shows the details of the included studies.

**Table 1:** The specifications of the included studies and their performance indicators

Studies	Age group	Gender	Algorithm	AUC (95% CIs)	Other performance indicators
Del, N.A., et al. [46]	25% emerging adults (18–29 years), 48% established adults (30–45 years), and 26% midlife/older adults (46 years and older)	Approximately 71.16% of the participants were female.	Logistic Regression	0.86 (0.76–0.95)	Accuracy: 0.6957, Balanced Accuracy: 0.7585, Recall/Sensitivity: 0.8824, Specificity: 0.6346, Precision: 0.4412, F1 Score: 0.5882
			Random Forest	0.83(0.7–0.941)	Accuracy: 0.7536, Balanced Accuracy: 0.7574, Recall/Sensitivity: 0.7647, Specificity: 0.7500, Precision: 0.5000, F1 Score: 0.6047
Papini, S., et al. (2023) [47]	The average age was 26.9 years.	94.7% were men.	Gradient Boosting Machine	0.74 (0.71–0.77)	In the top decile (highest risk), PPV is 16.0% (95% CI 14.2–17.7), and sensitivity (cumulative) reaches 100%. The range of log loss values in the development phase was 0.372–0.375.
Liu, Y., et al. [28]	majority under 25 years old (56.2%)	predominantly female participants (77.3%)	Neural network	0.893	The neural network model accurately predicted 90.0% of the outcomes. The external test set correctly identified and classified 70.5% of positive and 95.2% of negative samples. A precision of 95.2% in predicting adverse outcomes and an accuracy of 70.5% in predicting positive outcomes.
Cui, K., et al. [27]	age between 18–80 years	NS	Logistic Regression	0.91 (86.9–95.1)	Accuracy 69.44%; Sensitivity: 0.870 for the modeling group; 70.83% for the validation group; Specificity: 0.881 for the modeling group; 68.75% for the validation group. The Hosmer–Lemeshow test model fitting effect showed a value of *P*=0.785.
Gagnon‑Sanschagrin, P., et al. [34]	18–64 years	NS	Random Forest	0.75	
Tomas, C.W., et al. [11]	Study A: The average age was 42.17 years. Study B: The average age was 33.75 years.	Study A: male (70.9%); Study B: with a slight female majority (55.1%)	Extreme Gradient Boosting	Internal validation of all variables: 0.77 (0.57–0.92), external validation of all variables: 0.70 (0.62–0.78)	Internal validation all variables: Accuracy: 0.91, Precision: 0.80, Recall: 0.57, F1 score: 0.66. External validation all variables: Accuracy: 0.75, Precision: 0.39, Recall: 0.62, F1 score: 0.48.
			Extreme Gradient Boosting	Internal RFE variables: 0.9 (0.74–1), External RFE variables: 0.73 (0.65–0.8), Internal all variables: 0.83 (0.64–1), External all variables: 0.76 (0.68–0.84)	RFE variables internal: Accuracy: 0.93, Precision: 0.85, Recall: 0.85, F1 score: 0.85. RFE variables external: Accuracy: 0.74, Precision: 0.45, Recall: 0.70, F1 score: 0.54. All variables internal: Accuracy: 0.90, Precision: 0.83, Recall: 0.71, F1 score: 0.76. All variables external: Accuracy: 0.81, Precision: 0.57, Recall: 0.67, F1 score: 0.62.
			Extreme Gradient Boosting	Internal RFE variables: 0.64 (0.35–0.85), External RFE variables: 0.46 (0.41–0.51), Internal all variables: 0.71 (0.50–0.92), External all variables: 0.48 (0.41–0.54)	RFE variables internal: Accuracy: 0.64, Precision: 0.66, Recall: 0.57, F1 score: 0.61. RFE variables external: Accuracy: 0.51, Precision: 0.53, Recall: 0.90, F1 score: 0.67. All variables internal: Accuracy: 0.71, Precision: 0.80, Recall: 0.57, F1 score: 0.66. All variables external: Accuracy: 0.52, Precision: 0.54, Recall: 0.90, F1 score: 0.67.
Howe, E.S., et al. [33]	The average age of 38.25 years	60% male	Elastic net regularized regression	0.66 (0.5–0.89)	Average sensitivity 0.69, average Brier score 0.19, average specificity 0.69.
Schultebraucks, K., et al. (2021) [42]	Average age was 46.09 years	37.2% females, 62.8% males.	eXtreme Gradient Boosting	0.89 AUC multiclass	Accuracy = 0.79 (95% CI: 0.69–0.87), Precision = 0.83.
			eXtreme Gradient Boosting	0.89 (0.71–1)	Precision = 0.97. Sensitivity = 1.00 (95% CI: 1-1), Specificity = 0.81 (95% CI: 0.71–1).
Morris, M.C., et al. [32]	aged 18 to 30	Females	Gradient Boosting Machine	0.96	
			Logistic regression	0.91	
Gokten, E.S., et al. [41]	children and adolescents	NS	Random Forest	0.76	Accuracy: 0.72 (± 0.12), Precision 0.72 (± 0.12), Recall 0.71 (± 0.12), F1 score: 0.71 (± 0.12).
Schultebraucks, K., et al. (2020) [31]	ages between 18 and 70 years, average age 37.86 years	42.5% female	Neural network	0.9	weighted average precision = 0.83, recall = 0.84, and f1-score = 0.83
Wshah, S., et al. [36]	average age of 35 years	mostly male (57 out of 90)	Logistic regression	0.83	Accuracy: Train-Test: 0.8735, Cross-Validation: 0.8211.
			Naive Bayes	0.84	Accuracy: Train-Test: 0.8711, Cross-Validation: 0.8221
			SVM-linear kernel	0.82	Accuracy: Train-Test: 0.8349, Cross-Validation: 0.7641
			SVM-Gaussian kernel	0.85	Accuracy: Train-Test: 0.8633, Cross-Validation: 0.8191
			SVM-polynomial kernel	0.83	Accuracy: Train-Test: 0.8682, Cross-Validation: 0.8186
			Random forest	0.78	Accuracy: Train-Test: 0.8212, Cross-Validation: 0.7789
			Voting classifier-soft	0.85	Accuracy: Train-Test: 0.8799, Cross-Validation: 0.8205
			Voting classifier-hard	0.83	Accuracy: Train-Test: 0.8592, Cross-Validation: 0.8070
Zandvakili, A., et al. [35]	mean age 51.6 years	60% were male	SVM	0.71 (0.54–0.87)	
Papini, S., et al. (2018) [44]	NS	NS	eXtreme Gradient Boosting	Full model: 0.85 (0.83–0.86), Model with Hospital Features Only: 0.78 (0.76–0.80)	Full model: Sensitivity: 0.69 [95% CI 0.66–0.72], Specificity: 0.83 [95% CI 0.80–0.85], Positive Predictive Value: 0.65 [95% CI 0.62–0.69], Negative Predictive Value: 0.86 [95% CI 0.84–0.87], Overall Accuracy: 0.78 [95% CI 0.77–0.80]. Model with Hospital Features Only: Sensitivity: 0.51 [95% CI 0.48–0.55], Specificity: 0.87 [95% CI 0.86–0.88], Positive Predictive Value: 0.63 [95% CI 0.61–0.66], Negative Predictive Value: 0.80 [95% CI 0.79–0.81], Overall Accuracy: 0.76 [95% CI 0.74–0.77].
			Logistic Regression	0.75 (0.73–0.76)	Sensitivity: 0.57 [95% CI 0.53–0.61], Specificity: 0.76 [95% CI 0.73–0.79], Positive Predictive Value: 0.53 [95% CI 0.50–0.56], Negative Predictive Value: 0.80 [95% CI 0.79–0.81], Overall Accuracy: 0.70 [95% CI 0.68–0.72]
Nicholson, A.A., et al. [43]	NS	71% female	Multiclass Gaussian Process Classification	NS	a balanced accuracy of 91.63% for predicting PTSD, PTSD + DS, and healthy controls using mALFF maps. The class accuracy for healthy individuals was 96.08%, for PTSD patients 89.02%, and for PTSD + DS patients 89.80%. The predictive class value (akin to precision) for healthy individuals was 87.50%, for PTSD patients 94.81%, and for PTSD + DS patients 89.90%.
Rosellini, A.J., et al. [45]	NS	NS	Super learner	0.79 (0.78–0.8)	MSE 9.94
			Logistic full set	0.77 (0.76–0.78)	MSE 10.25
			Logistic Lasso set	0.77 (0.76–0.78)	MSE 10.25
			Logistic T-test set	0.77 (0.76–0.78)	MSE 10.31
			Elastic net full set (MPP=0.25)	0.77 (0.76–0.78)	MSE 10.23
			Elastic net Lasso set (MPP=0.25)	0.77 (0.76–0.78)	MSE 10.24
			Elastic net T-test set (MPP=0.25)	0.76 (0.76–0.77)	MSE 10.31
			Elastic net full set (MPP=0.50)	0.7733 (0.7654–0.7812)	MSE 10.22
			Elastic net Lasso set (MPP=0.50)	0.7727 (0.7648–0.7807)	MSE 10.25
			Elastic net T-test set (MPP=0.50)	0.7652 (0.7571–0.7734)	MSE 10.31
			Elastic net full set (MPP=0.75)	0.773 (0.7651–0.781)	MSE 10.24
			Elastic net Lasso set (MPP=0.75)	0.7727 (0.7648–0.7808)	MSE 10.25
			Elastic net T-test set (MPP=0.75)	0.7652 (0.7571–0.7733)	MSE 10.3
			Lasso Full set	0.7729 (0.765–0.7809)	MSE 10.24
			Lasso Lasso set	0.7727 (0.7648–0.7806)	MSE 10.25
			Lasso T-test set	0.7652 (0.757–0.7733)	MSE 10.3
			Adaptive splines full set	0.7767 (0.7687–0.7846)	MSE 10.17
			Adaptive splines Lasso set	0.7772 (0.7693–0.7852)	MSE 10.16
			Adaptive splines T-test set	0.771 (0.7629–0.7791)	MSE 10.22
			Adaptive polynomial splines full set	0.7364 (0.7279–0.7449)	MSE 10.56
			Adaptive polynomial splines Lasso set	0.7496 (0.7412–0.7581)	MSE 10.45
			Adaptive polynomial splines T-test set	0.7619 (0.7538–0.7701)	MSE 10.35
			Random Forest full set	0.7766 (0.7686–0.7847)	MSE 10.15
			Random Forest Lasso set	0.7703 (0.7621–0.7785)	MSE 10.29
			Random Forest T-test set	0.723 (0.7084–0.7376)	MSE 11.17
			Bayesian Adaptive Trees full set	0.7836 (0.7759–0.7913)	MSE 10.07
			Bayesian Adaptive Trees Lasso set	0.7836 (0.7759–0.7913)	MSE 10.07
			Bayesian Adaptive Trees T-test set	0.779 (0.7711–0.7868)	MSE 10.09
			SVM (Linear) full set	0.5269 (0.516–0.5379)	MSE 11.57
			SVM (Linear) Lasso set	0.5186 (0.5078–0.5295)	MSE 11.53
			SVM (Linear) T-test set	0.5859 (0.5753–0.5965)	MSE 11.43
			SVM (Polynomial) full set	0.6442 (0.633–0.6554)	MSE 11.22
			SVM (Polynomial) Lasso set	0.632 (0.6207–0.6434)	MSE 11.28
			SVM (Polynomial) T-test set	0.5021 (0.4868–0.509)	MSE 11.54
			SVM (Radial) full set	0.686 (0.6759–0.6962)	MSE 10.91
			SVM (Radial) Lasso set	0.6757 (0.6651–0.6862)	MSE 10.97
			SVM (Radial) T-test set	0.5627 (0.5518–0.5736)	MSE 11.5
Zhu, Z., et al. [40]	between the ages of 18 and 65 years	68% female	Deep learning	NS	average accuracy of 80%, average sensitivity of 80.9%, and specificity of 79.2%.
			SVM	NS	average accuracy of 57.7%, average sensitivity of 53.2%, and specificity of 62.2%.
McDonald, A.D., et al. [38]	ranged from 24 to 74 years, with an average age of 47.3 years.	NS	SVM	0.67	NS
			Decision Tree	0.61	NS
			Neural network	0.6	NS
			Random Forest	0.66	NS
			CNN	0.63	NS
Li, Y., et al. [30]	Average age 23.2	69.5% were male	Light Gradient Boosting Machine model with DART (Dropouts meet Multiple Additive Regression Trees) boosting method	training folds 0.99 (SD= 0.002), testing folds 0.93 (SD= 0.04)	At the point with the highest Youden’s index, the sensitivity was 0.908, and the precision was 0.260. At the threshold selected for best overall performance, the sensitivity was 0.862, and the precision was 0.272.
Worthington, M.A., et al. [48]	NS	NS	Bayesian Additive Regression Trees	0.95	Sensitivity: 97.7%, specificity: 67.7%
			Penalized Logistic Regression	0.92	Sensitivity: 93.3%, specificity: 55.8%
			Classification Trees	0.92	Sensitivity:92.0%, specificity: 0.0%
Ziobrowski, H.N., et al. [39]	aged between 18 and 75 years	67.9% females	An ensemble machine learning model	0.81	mean Integrated Calibration Index of 0.040 with SE 0.002; mean Expected Calibration Error of 0.039 with SE 0.002
Marmar, C.R., et al. [37]	adults	Males	Random Forest	0.95	Overall correct classification rate: 89.1%, Youden’s index: 0.787.
Ucuz, I., et al. [29]	children and adolescents	88% females	Neural network		Average accuracy across all systems was found to be 99.2%.

PTSD: post-traumatic stress disorder, NS: not specified; MSE: mean square error

### Risk of bias

**Fig. 2:**
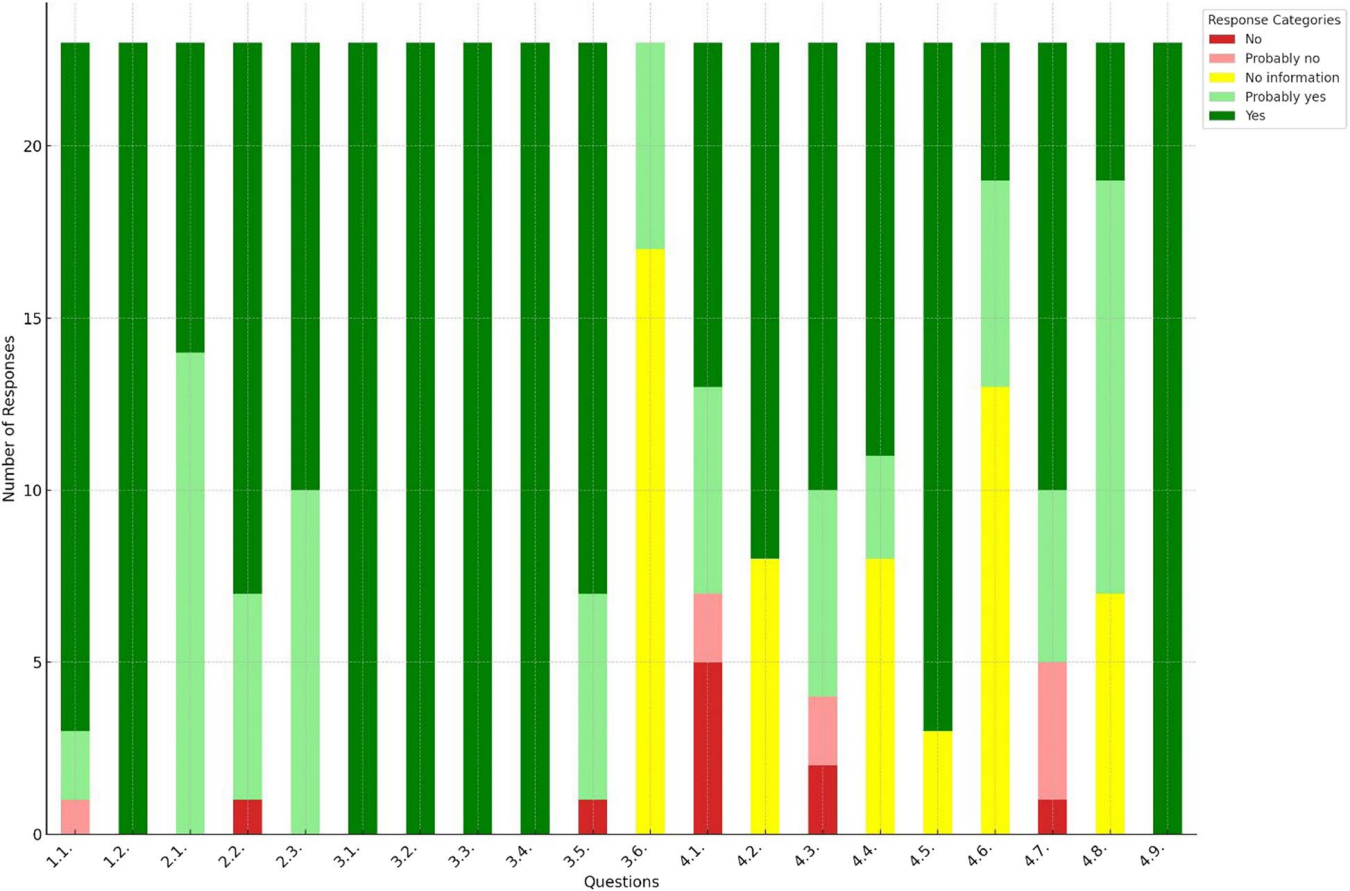
The assessment of the risk of bias of the studies across 20 PROBAST questions. Where: (1.1) Were appropriate data sources used, e.g., cohort, RCT, or nested case–control study data? (1.2) Were all inclusions and exclusions of participants appropriate? (2.1) Were predictors defined and assessed in a similar way for all participants? (2.2) Were predictor assessments made without knowledge of outcome data? (2.3) Are all predictors available at the time the model is intended to be used? (3.1) Was the outcome determined appropriately? (3.2) Was a prespecified or standard outcome definition used? (3.3) Were predictors excluded from the outcome definition? (3.4) Was the outcome defined and determined in a similar way for all participants? (3.5) Was the outcome determined without knowledge of predictor information? (3.6) Was the time interval between predictor assessment and outcome determination appropriate? (4.1) Were there a reasonable number of participants with the outcome? (4.2) Were continuous and categorical predictors handled appropriately? (4.3) Were all enrolled participants included in the analysis? (4.4) Were participants with missing data handled appropriately? (4.5) Was the selection of predictors based on univariable analysis avoided? (4.6) Were complexities in the data (e.g., censoring, competing risks, sampling of control participants) accounted for appropriately? (4.7) Were relevant model performance measures evaluated appropriately? (4.8) Were model overfitting, underfitting, and optimism in model performance accounted for? (4.9) Do predictors and their assigned weights in the final model correspond to the results from the reported multivariable analysis?

The assessment of 23 articles using the PROBAST tool reveals that for the participant questions (data source and inclusion/exclusion criteria), there was a unanimous agreement on the adequacy of participant selection, with most articles receiving a “yes” or “probably yes.” Predictor questions (2.1 to 2.3) received “probably yes” and “yes” responses, indicating generally acceptable predictor handling. For outcome-related questions (3.1 to 3.6), responses were consistently “yes,” except for question 3.6, where “no information” was the majority response, indicating a lack of detail in reporting. In the analysis domain (4.1 to 4.9), responses varied more, with a mix of “yes,” “probably yes,” and “no information.” The final question (4.9) saw a unanimous “yes,” reflecting consistency across the studies’ final analysis. Please go to Fig. 2 for further details.

**Fig. 3:**
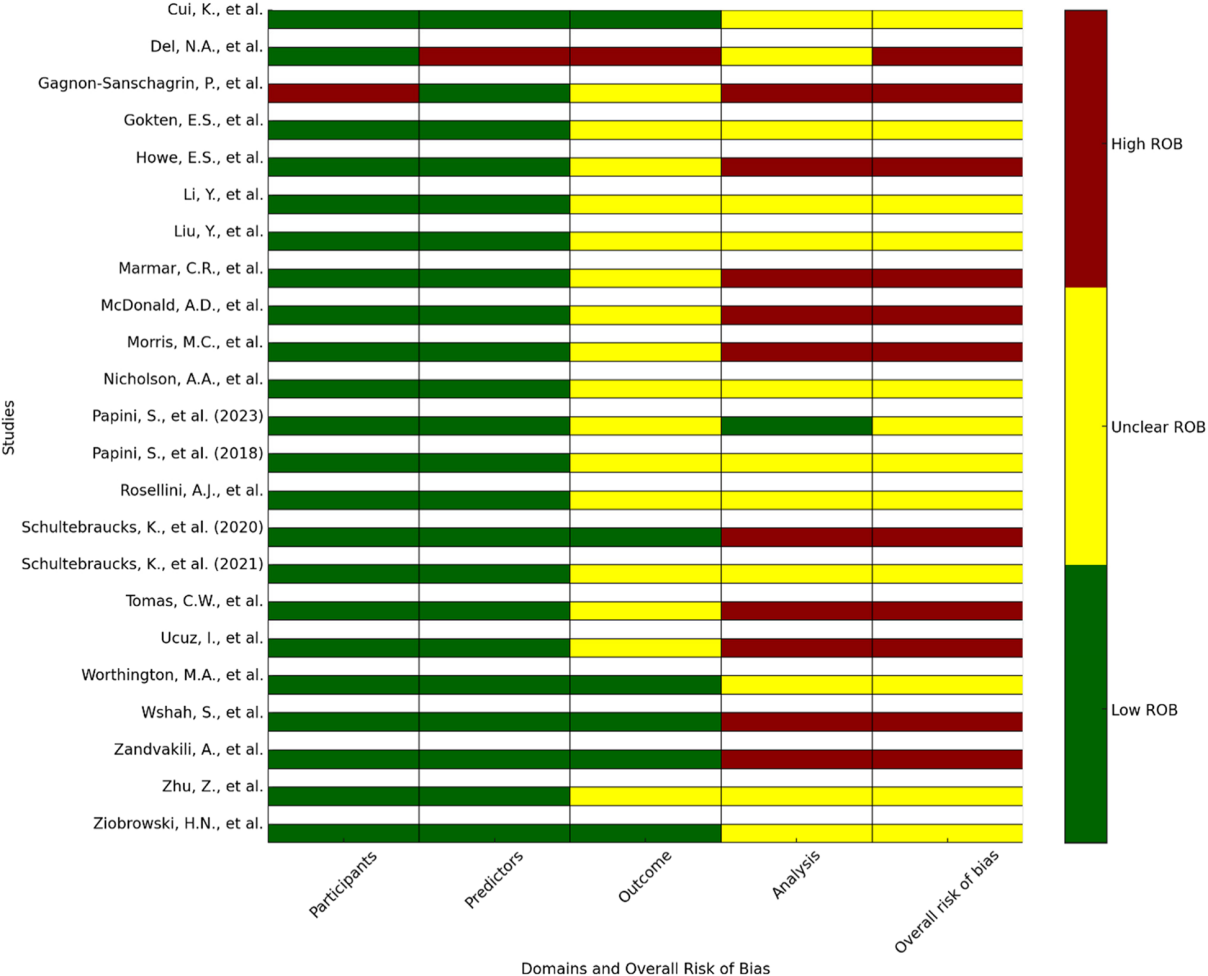
The risk of bias in the studies in terms of PROBAST domains and ROB overall

The Risk of Bias (ROB) was conducted in four domains for individual studies: participants, predictors, outcome, analysis, and the overall ROB. As for the domain of participants, the risk of bias was low, with 96% (*n* = 22) of the articles showing low ROB, while only 4% (*n* = 1) had reached a high ROB [34]. No uncertainty was reported in this domain. In the predictors’ domain, the results were identical to the participants’ domain, with 96% (*n* = 22) of studies presenting a “low” risk and 4% (*n* = 1) a “high” risk [46], reinforcing the robustness of predictor variables in the evaluated research. The outcome domain presented more varied results, with most studies (70%, *n* = 16) exhibiting an “unclear” risk of bias [11, 28–30, 32–34, 37, 38, 40–45, 47], while 26% (*n* = 6) were assessed as “low” risk [27, 31, 35, 36, 39, 48]. , and a minimal number (4%, *n* = 1) as “high” risk [46]. Analysis was the domain with the highest observed risk, where 43% (*n* = 10) of the articles were classified as “high” ROB [11, 29, 31–38], and a substantial proportion (52%, *n* = 12) were rated as “unclear.” [27, 28, 30, 39–46, 48]. Only one study (4%) [47] was judged to have a “low” risk of bias in this domain. Overall, the ROB for the collected articles revealed that 48% (*n* = 11) of them exhibited a “high” ROB [11, 29, 31–38, 46], and 52% (*n* = 12) were judged as “unclear.” [27, 28, 30, 39–45, 47, 48], with none of the articles being scored as “low” risk, indicating a substantial degree of uncertainty and potential bias within the reviewed studies, emphasizing the necessity for more rigorous methodological standards and transparent reporting to enhance the validity and reliability of research findings in this field. Please refer to Fig. 3 for further information.

### Performance of different types of algorithms in various populations

Meta-analytical results from various studies suggest the superiority of tree-based models over neural network models in predicting (PTSD among individuals with war and military experiences. Specifically, tree-based models achieved a pooled AUC of 0.745 (95% CIs 0.572–0.917, I^2^ = 97.31%), compared to 0.615 (95% CIs 0.552–0.768, I^2^ = 0%) for neural networks. Additionally, only one study applied a SVM model in this demographic, yielding an AUC of 0.67 (95% CIs 0.59–0.75) [38]. External validation of a tree-based model in a study within this group indicated an AUC of 0.74 (95% CIs 0.71–0.77) [47]; however, the limited number of studies precluded further meta-analytical efforts.

For individuals with experiences of sexual or physical trauma, the employment of tree-based models resulted in a pooled AUC of 0.861 (95% CIs 0.723–1, I^2^ = 96.78%). A linear model in this context revealed an AUC of 0.91 (95% CIs 0.83–0.99) [32], though the scarcity of similar studies hindered a meta-analysis. Regarding natural disasters, meta-analysis encompassed three algorithms: linear models, SVM, and tree-based models. Tree-based models led with a pooled AUC of 0.771 (95% CIs 0.755–0.787, I^2^ = 90.41%), closely followed by linear models with an AUC of 0.768 (95% CIs 0.764–0.771, I^2^ = 78.76%). On the other hand, SVM models showed the most minor efficacy, with a pooled AUC of 0.593 (95% CIs 0.55–0.636, I^2^ = 99.39%). A super learner algorithm with an AUC of 0.79 (95% CIs 0.78–0.80) was also reported in a study [45]. However, we did not conduct a meta-analysis of this model because of the insufficient number of AUCs available. Additionally, deep learning was applied in another study to predict PTSD in populations affected by natural disasters, but the AUC was not reported in this study [40].

In the context of medical trauma, analyses were conducted for two algorithms, revealing pooled AUCs of 0.828 (95% CIs 0.717–0.939, I^2^ = 98.18%) for linear models and 0.808 (95% CIs 0.761–0.855, I^2^ = 95.25%) for tree-based models. External validation of tree-based models in this demographic reported a pooled AUC of 0.591 (95% CIs 0.47–0.712, I^2^ = 93.61%). The meta-analysis of neural networks and ensemble methods in this population was impossible because of a lack of AUCs, with a reported AUC of ensemble method 0.79 (95% CIs 0.78–0.8) [45]. The AUC of neural network in another study of this population was not reported [40].

In general PTSD populations, the meta-analysis included SVM, ensemble methods, and linear models, recording pooled AUCs of 0.824 (95% CIs 0.778–0.871, I^2^ = 0%), 0.841 (95% CIs 0.783–0.898, I^2^ = 0%), and 0.768 (95% CIs 0.608–0.928, I^2^ = 60.06%), respectively. Limited AUC availability constrained the meta-analysis of Bayesian and tree-based methods in this population, as reported in a study [36].

**Fig. 4:**
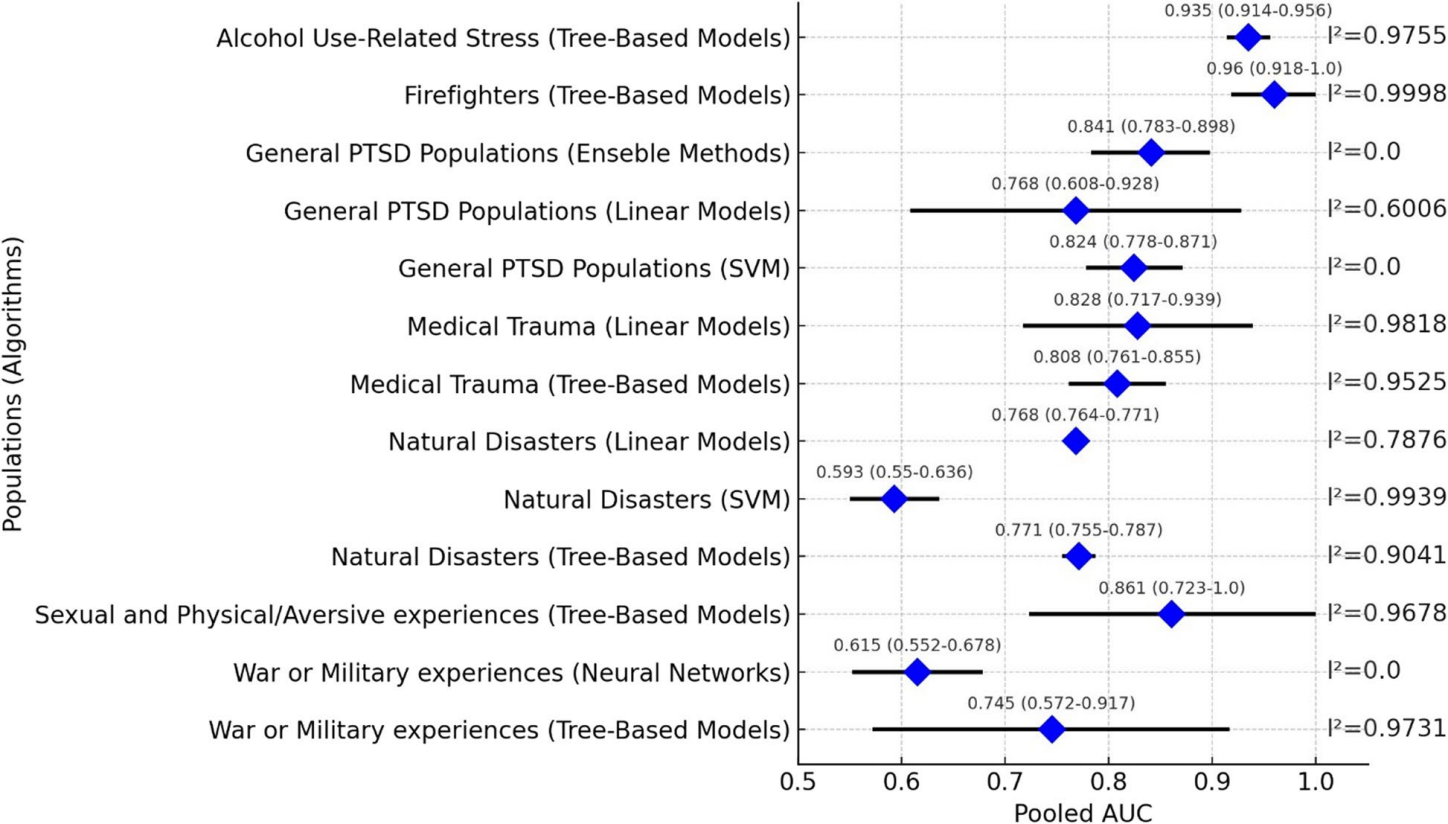
Forest plot of pooled internal AUCs in different populations and algorithms

Among firefighters, tree-based models achieved the highest pooled AUC across all examined demographics, at 0.96 (95% CIs 0.918–1, I^2^ = 99.98%). Similarly, in populations with alcohol-related stress, these models produced a pooled AUC of 0.935 (95% CIs 0.914–0.956, I^2^ = 97.55%). In insured adults, only one study utilizing a random forest model was available, with a reported AUC of 0.75 (95% CIs 0.747–0.752) [34]; consequently, a meta-analysis could not be conducted. Figure 4 displays the pooled internal AUCs of different algorithms across various populations.

Publication bias was assessed in two meta-analyses: one in general PTSD populations (linear models) and the other in medical trauma (tree-based models), as the other meta-analyses had an insufficient number of studies. The results of the Egger test in both meta-analyses indicated no publication bias (P-value > 0.05). Sensitivity analyses revealed a significant reduction in heterogeneity in three meta-analyses, including linear-based models in natural disasters (reducing I² from 78 to 57%), tree-based models in natural disasters (reducing I² from 90 to 41.4%), and tree-based models in war populations (reducing I² from 97 to 0%).

## Discussion

We conducted a systematic review and meta-analysis of machine learning studies to predict PTSD across diverse populations. A total of 23 studies were included in the systematic review. The studied populations included the following: War and military engagement experiences, sexual and physical/aversive trauma, natural disasters, pandemic-related stress, medical trauma, general PTSD, insured adults, firefighters, and alcohol-related stress. In each group, we conducted meta-analyses using different types of algorithms.

Tree-based methods were the main algorithms used in the studies to predict PTSD. We could conduct meta-analyses of tree-based methods in all the populations exempt from general PTSD populations. The results obtained from the present study showed that tree models generally performed better in prediction than other models, regardless of architecture or features. This result can be observed in some populations, such as those affected by alcohol consumption, sexual harassment, or war or military experiences. Nevertheless, this evidence overlooks the significant or ambiguous risk of bias inherent in all studies and the outcomes from external validation. The main contributors to the high risk of bias in the studies were small sample sizes, including all participants in the final analysis, and evaluating model performance. The substantial variability in clinical outcomes across studies complicates the comparison of performance. This variability can be attributed to differences in the timelines of outcomes, characteristics of the research population, predictive factors, and model architectures [16]. On the other hand, training machine learning models unavoidably involve a considerable amount of heterogeneity [49, 50]. High heterogeneity in meta-analyses can often be attributed to the variability in the models used across studies, as well as the specific types of populations examined. Differences in methodological approaches, such as the predictive variables included in the models and the demographic characteristics of the populations (e.g., age, gender, and comorbid conditions), can lead to substantial variation in effect sizes. This variability is a primary reason for the observed high heterogeneity in our analysis, as it reflects the complex nature of PTSD prediction across diverse contexts.

Only two articles conducted external validation, which is essential for reliable comparisons. The lack of external validation remains a persistent methodological problem in research employing machine learning and deep learning techniques [51–53], and the current evidence confirms that validation by independent researchers is uncommon [54], and only a small number of models undergo external validation [49]. Tree-based models were the only algorithm used in the studies that included external validation. When examining the pooled AUC from external validations in medical trauma and the single AUC from a survey focused on war and military experiences, external validation showed poorer results than internal validation. This may be explained by the following reasons: Tree-based models are prone to overfitting the training data, capturing noise as if it were a signal. These results in high performance in internal validation but poor generalization to new datasets [55]. If the external validation data differs extensively in distribution from the training data, the model will represent unacceptable accuracy [56]. Complex models that employ numerous parameters may also become overly fitted to the training data and undermine their performance on externally varied datasets [57].

Some of the included studies exhibited a high risk of bias attributable to small sample sizes. In PTSD prediction, the dataset size is more than just a number [10]; it also affects the quality of the data [57]. Inadequate sample size can lead to overfitting, where models perform well on training data but fail on new, unseen data [58]. This issue is particularly alarming in clinical contexts such as PTSD prediction, where the imperative is the precise identification of individuals requiring intervention [10]. Consequently, this may result in biased predictive models and may demonstrate low efficacy in clinical environments. In a study that assessed the risk of bias in prediction models for adults with heart failure, the results indicated significant biases in the studies because of the sample size [59]. Furthermore, similar results were identified in another study that evaluated the risk of bias in prediction models developed using supervised machine-learning techniques [9].

Excluding some participants from the final analysis contributed to a significant risk in some studies. Attrition bias can happen for various reasons, such as participants losing contact, withdrawing, or being excluded because of incomplete data. In machine learning development, this selective inclusion of data can negatively affect the training of models. Therefore, the model’s accuracy may be reduced, leading to unreliable predictions in real-world settings [60]. Attrition bias was also identified as a source of risk of bias in studies on prediction models developed employing supervised machine learning techniques [9]. Some included studies also failed to report the performance of their models. Ethically, it is essential to document the performance of machine learning models with different indicators because of transparency and to ensure their efficacy in real-world data [61]. Inaccurate or incomplete reporting can result in misinformed decisions, especially in fields that directly impact human lives. Therefore, machine learning studies related to PTSD prediction must disclose all performance indicators and adhere to established reporting standards in machine learning. It was found that most studies did not report the racial description and information of the populations. Discrimination based on race acts as a stress-inducing factor, influencing how individuals respond to traumatic events. The available evidence implies a link between racial bias and the development of symptoms associated with PTSD [62, 63]. Hence, it is crucial to conduct in-depth examinations that capture the complete demographic profile of the entire cohort in PTSD prediction studies.

The best prediction models were tree-based models for populations related to alcohol use and firefighters, showing the appropriateness of tree-based models in these populations. In populations affected by natural disasters, linear and tree-based models presented more accurate models than SVM models, showing their superiority over SVM in this context. However, the risk of bias, high heterogeneity, and lack of external validation of the included studies limit the interpretation of results. The use of various supervised machine learning algorithms for disease prediction was investigated in a study. The findings revealed that the SVM algorithm was the most commonly used, followed by the Naïve Bayes algorithm. The Random Forest (RF) algorithm also demonstrated the highest accuracy compared to other models [64]. Recent research focused on the effectiveness of machine learning and deep learning models in predicting long-term outcomes for patients with chronic obstructive pulmonary disease (COPD). The findings reveal moderate predictive accuracy for both exacerbation and mortality risks, with AUC statistics around 0.77, though these models did not significantly outperform existing disease severity scores [16]. In another similar article, the efficacy of some machine learning algorithms in predicting ischemic heart disease (IHD) was studied. The study showed the excellent performance of specific machine learning models. The XGBoost model demonstrated high accuracy with an AUC of 0.98. Moreover, the CatBoost model showed high predictive performance, with an AUC of 0.87. Other models, like logistic regression and SVM, were also introduced with AUCs of 0.963 and 0.76, respectively [65].

Implementing the models in a clinical setting is also greatly important [66]. Consider a practitioner who wants to use a model to predict a patient’s PTSD. If the model requires, for instance, 50 or more predictors, it may pose challenges in actual clinical practice. Therefore, in such cases, practitioners might prefer to use traditional PTSD risk assessment tools, such as the PCL-5 [67]. The selection and use of predictors and features in models for predicting PTSD across different populations should be a focus of future research. For instance, it would be valuable to examine which algorithm achieves the highest accuracy in predicting PTSD using data on electrical activity [68] or heart rate [69] in specific populations. Finally, forecasting diseases remains challenging, and there is no certainty that machine learning models will successfully predict patient outcomes, even though machine learning holds significant promise. Internal and external validation studies are insufficient to confirm the therapeutic effectiveness of these models [16]. Investigating the impact of these models on patient outcomes may necessitate conducting interventional trials, subtle interventions, and impact assessments [70].

To the best of our knowledge, this study is the first meta-analysis aimed at comparing machine learning algorithms for predicting PTSD across all populations. In our systematic review and meta-analysis, we searched three significant databases to identify relevant studies. Moreover, the PROBAST tool was employed to assess the risk of bias in the included studies. Furthermore, we utilized meta-analysis, a robust method for synthesizing evidence, to identify the most accurate model by pooling the AUCs of similar algorithms. The screening, study selection, data extraction, and risk of bias assessments were conducted independently by two reviewers to enhance the study’s quality. On the other side, a significant limitation of our study is the high heterogeneity among the included studies, which limits the interpretability of the results. Moreover, excluding non-English literature may overlook relevant studies and impact the results of this review. Although we searched in three of the most comprehensive databases, including PubMed, Scopus, and Web of Science, we did not search other databases such as EMBASE and the Cochrane Library. Consequently, some eligible studies may have been overlooked. Our study did not explicitly analyze how PTSD outcomes may differ across various trauma types, which could affect model performance. Additionally, the performance of machine learning algorithms varied based on the indicators used (e.g., AUC, accuracy), complicating direct comparisons. Future research should address these aspects for a more nuanced understanding of PTSD prediction. In some cases, we observed that the models included in the meta-analysis originated from a single study but employed different variables and settings. This variation may contribute to the high heterogeneity observed in some meta-analyses. Therefore, we suggest that future research should consider meta-regression when sufficient and homogeneous data become available.

## Conclusions

Overall, the best performance in terms of PTSD outcome prediction was shown by tree-based models. However, the evidence from these studies proved highly limited due to several factors, such as high heterogeneity, high and unclear risk of bias, and the need for more external validation models in the studies. Tree-based models tend to perform very well across various populations, particularly in those with particular trauma types such as alcohol use-related stress or firefighters, despite the high heterogeneity, indicating the need for careful model selection and tuning specific to each study. Linear and ensemble methods are more consistent and sometimes more effective in more generalized populations. High heterogeneity in many meta-analyses suggests that while a specific model type might be effective on average, its performance can vary greatly, indicating that contextual factors, such as the specifics of the dataset and model tuning, play critical roles. To enhance the quality of future research, it is recommended that researchers adhere to AI/ML reporting and PROBAST guidelines. Furthermore, researchers in this field should prioritize the external validation of these models to confirm their effectiveness and applicability.

## Supplementary Information

Supplementary Material 1.

## Data Availability

Data sharing is not applicable to this article, as no new datasets were generated or analyzed during this study. The data supporting the findings of this study are derived from previously published articles, which are duly cited. Further information can be provided by the corresponding author upon reasonable request.

## Abbreviations

NS: Not Specified
AUC: Area Under the Curve
PTSD: Post-Traumatic Stress Disorder
SVM: Support Vector Machine
CI: Confidence Interval
ML: Machine Learning
