# Supplementary material for: Machine learning algorithms for predicting PTSD: a systematic review and meta-analysis
Source: BMC Med Inform Decis Mak. 2025 Jan 21;25:34. doi: 10.1186/s12911-024-02754-2 (PMC11752770; doi:10.1186/s12911-024-02754-2)
Supplement: Supplementary file 1 — Supplementary Material 1. [file 12911_2024_2754_MOESM1_ESM.pdf]

S1. Search strategies

No.	Electronic database	Search formula
1	PubMed	(("Posttraumatic stress disorder"[Title] OR "post traumatic stress disorder"[Title] OR "post traumatic stress disorder"[Title]) AND ("Machine Learning"[MeSH Terms] OR "Machine Learning"[Title/Abstract] OR "Artificial Intelligence"[MeSH Terms] OR "Artificial Intelligence"[Title/Abstract] OR "predict*"[Title/Abstract] OR "prognos*"[Title/Abstract])) AND (2018:2024[pdat])
2	Scopus	TITLE ("Posttraumatic stress disorder" OR "post traumatic Stress Disorder" OR "post-traumatic stress disorder") AND TITLE-ABS-KEY ("Machine Learning" OR "Artificial Intelligence" OR predict* OR prognos*) AND PUBYEAR > 2017 AND PUBYEAR < 2025 AND (LIMIT-TO (DOCTYPE , "ar")) AND (LIMIT-TO (LANGUAGE , "English")) AND (LIMIT-TO (SRCTYPE , "j"))
3	Web of Science	"Posttraumatic stress disorder" OR "post traumatic Stress Disorder" OR "post-traumatic stress disorder" (Title) AND "Machine Learning" OR "Artificial Intelligence" OR predict* OR prognos* (Topic) and 2018 or 2019 or 2020 or 2021 or 2022 or 2023 (Publication Years) and Article (Document Types) and English (Languages)
